# Knowledge of HPV and HPV vaccine and its association with vaccination willingness among female undergraduates: A comparative cross-sectional study between healthcare and non-healthcare majors in Chengdu, China

**DOI:** 10.1371/journal.pone.0349013

**Published:** 2026-05-26

**Authors:** Miao Zhao, Jing Lei, Qin Feng, Shan Lai, Qiong Liang, Min Xie

**Affiliations:** 1 College of Pharmacy, Chengdu Medical College, Chengdu, Sichuan, China; 2 Department of Cosmetic Plastic and Burn Surgery, The First Affiliated Hospital of Chengdu Medical College, Chengdu, Sichuan, China; University of Naples Federico II: Universita degli Studi di Napoli Federico II, ITALY

## Abstract

**Purpose:**

This study aimed to assess levels of knowledge about human papillomavirus (HPV) and its vaccine among female undergraduates compare these levels between healthcare and non-healthcare majors, and examine their associations with vaccination willingness.

**Methods:**

A cross-sectional survey was conducted among 1,293 female undergraduate students from two universities in Chengdu. Knowledge was measured using a self-designed questionnaire, categorized into basic and advanced domains. Vaccination willingness was assessed via a 5-point Likert scale. Data were analyzed using descriptive statistics, t-tests, analysis of variance and hierarchical multiple regression to identify associations and compare the two student groups.

**Results:**

A total of 1191 valid questionnaires were analyzed. Participants demonstrated moderate basic knowledge of HPV (mean score = 5.21 ± 1.91/8) and its vaccine (mean score = 2.99 ± 1.05/5), but low advanced knowledge of HPV (mean score = 1.02 ± 1.03/6) and its vaccine (mean score = 2.00 ± 1.53/6). Students in healthcare majors demonstrated significantly higher scores in HPV basic knowledge (p = 0.023),HPV vaccine basic knowledge (p = 0.012), and advanced HPV vaccine knowledge (p < 0.001) compared to their non-healthcare peers. Overall vaccination willingness was higher among healthcare majors (p = 0.011), students from urban areas (p < 0.001), and those with higher monthly living expenses (p < 0.001). Hierarchical multiple regression analysis revealed that higher scores in basic knowledge were significantly associated with greater vaccination willingness (p < 0.001, △R^2^ = 0.127).

**Conclusions:**

The study confirms a significant knowledge and intention gap between healthcare and non-healthcare students. Basic knowledge of HPV and its vaccine acts as an effective peripheral cue that boosts vaccination willingness, aligning with the Elaboration Likelihood Model (ELM). However, widespread deficits in advanced knowledge, even among healthcare students, impede central-route processing and durable attitude formation. Public health efforts should prioritize clear core messaging while simplifying and disseminating advanced knowledge to narrow knowledge gaps across student students with different academic backgrounds.

## Introduction

Cervical cancer remains a leading cause of cancer-related mortality among women worldwide. In 2022, cervical cancer was the fourth most commonly occurring cancer among women globally and also the fourth leading cause of cancer deaths among women, accounting for around 662 000 new cases and around 349 000 deaths [1]. In China, cervical cancer holds the position of the sixth most prevalent form of cancer among women [2]. Almost all cervical cancer cases are linked to infection with Human papillomaviruse (HPV). About 99.7% of cervical cancer cases are caused by persistent genital high-risk HPV [3]. Despite most HPV infections resolving spontaneously without causing any symptoms, persistent infection can lead to cervical cancer in women [3]. In addition, HPV infection may also cause anal cancer, oropharyngeal cancer, vaginal cancer, etc. [4].

HPV vaccination has proven to be a highly effective primary prevention method in many countries, significantly reducing the incidence of cervical and other HPV-related cancers [5]. A study also confirmed that switching from bivalent or quadrivalent (first generation HPV vaccines) to (second-generation) nonavalent HPV vaccines can greatly improve the prevention rate of cervical lesions [6]. As a voluntary health behavior, the uptake of HPV vaccine largely depends on people’s knowledge and attitudes of HPV and HPV vaccination [7,8]. Numerous studies have reported positive associations between knowledge of HPV and HPV vaccine and vaccination willingness [9–14]. However, the level of knowledge is not uniformly distributed across populations and can be profoundly shaped by socio-demographic and educational contexts.

Related study shows that HPV infection peaks among Chinese women occurs between the ages of 20 and 24 [15], a time when many are attending college or university. Therefore, female university students, a group often in the age range of high HPV exposure risk and a key target for catch-up vaccination [16–18], present a crucial population for investigation. Their knowledge levels and vaccination willingness may vary significantly based on their academic background. Extensive research across different educational contexts has established that students in health- and bioscience-related disciplines (including medical, biology, and other health-related majors) consistently outperform their peers in unrelated fields in terms of HPV knowledge and vaccination attitudes [12,19–32]. Despite this well-documented pattern, significant gaps persist in the literature. First, a common limitation is the treatment of HPV knowledge as a unitary construct, failing to distinguish between basic and advanced dimensions, which obscures their potentially distinct roles. Second, the specific drivers of the intention gap within the unique environment of a university campus in China warrant focused investigation, considering local cultural and socio-economic factors.

Therefore, this study aims to conduct a comparative cross-sectional analysis to elucidate the differences in HPV and HPV vaccine knowledge and their association with vaccination willingness between female undergraduates in healthcare and non-healthcare majors. Specifically, our objectives are: (1) to assess and compare the levels of basic and advanced knowledge of HPV and its vaccine between the two student groups; (2) to evaluate and compare their willingness to receive the HPV vaccine; and (3) to examine how different dimensions of knowledge are associated with vaccination willingness within each group. The findings from this study will provide an evidence base for developing more effective health education programs to enhance vaccination willingness and increase HPV vaccine uptake among female university students.

## Materials and methods

### Study design

This cross-sectional study was conducted from March to July 2024 at an independent medical college and a comprehensive university in Chengdu, Sichuan, China, using an electronic questionnaire to collect data. The target population comprised female undergraduate students enrolled at these two institutions during the study period. This study adhered to the Strengthening the Reporting of Observational Studies in Epidemiology (STROBE) guidelines.

### Sampling and participants

Students from each institution were sampled using stratified sampling by academic year and major. During the sampling process, efforts were made to achieve a relatively balanced representation across all undergraduate year levels, as well as between students majoring in healthcare and non-healthcare disciplines. Healthcare majors were selected from four programs at the medical college: Nursing, Clinical Medicine, Preventive Medicine, and Pharmacy. Non- healthcare majors were randomly selected from four programs at the comprehensive university.

According to the literature survey, the estimated sample size was determined based on the following formula:


$$\begin{array}{c} n=\frac{\overset{2}{\underset{\alpha /2}{Z}}\times \text{p}\left(\text{1-p}\right)}{{\text{d}}^{2}} \end{array}$$

where d is the permissible error and takes the value of d = 0.03, p is the anticipated proportion of female college students willing to receive the HPV vaccine. Recent meta-analyses [33,34] conducted in China reported that 68.0–71.8% of Chinese female college students expressed willingness to be vaccinated against HPV; The estimation was based on a 95% confidence level (α = 0.05, Z_α/2_ = 1.96).therefore, we used p = 0.70 for sample size calculation. The final sample size was at least 897. To account for potential invalid questionnaires, the sample size was increased by 15%, leading to a minimum required sample size of 1032 participants.

Participants were eligible for inclusion if they met all of the following criteria: (1) Chinese female undergraduate students enrolled in their first to third academic year; (2) had heard of HPV and the HPV vaccine; (3) informed consent and voluntary participation in this study. The exclusion criteria were: (1)students who had already received the HPV vaccine; (2) not willing to participate in this study.

### Instruments

The following survey instruments were administered: a demographic characteristics questionnaire, an HPV Knowledge Questionnaire, an HPV Vaccine Knowledge Questionnaire, and an HPV Vaccination Willingness Scale.

#### Demographic characteristics questionnaire.

The demographic characteristics questionnaire collected information on the following variables: age, academic major (categorized as healthcare or non-healthcare), academic year (1st, 2nd or 3rd year), Residence (urban or rural), monthly living expenses (in Chinese Yuan/ CNY), sexual history (yes/no).

#### HPV knowledge questionnaire and HPV vaccine knowledge questionnaire.

The HPV knowledge questionnaire and HPV vaccine knowledge questionnaire both consisted of two domains: basic knowledge and advanced knowledge. Previous studies have primarily employed true/false questions [9–11,17,19,20,24,28,29,35–47]. To more accurately reflect the respondents’ knowledge level, this study utilizes multiple-choice questions, comprising both single-answer and multiple-answer types.

Single-answer questions presented four response options, including one “I don’t know” option. Only one option was correct. A correct selection was awarded 1 point; incorrect selections or selection of “I don’t know” received 0 point. Multiple-answer questions presented five response options, including one “I don’t know” option. Each question had more than one correct answer. A full point (1 point) was awarded only if all and only the correct options were selected; incorrect selection, omission of a correct option, selection of any incorrect option, or selection of “I don’t know” received 0 point.

The HPV basic knowledge domain comprised 7 single-answer and 1 multiple-answer question, while the HPV advanced knowledge domain included 3 single-answer and 3 multiple-answer questions. Total knowledge scores ranged from 0 to 14. The HPV vaccine basic knowledge domain comprised 4 single-answer and 1 multiple-answer question, while the HPV vaccine advanced knowledge domain included 3 single-answer and 3 multiple-answer questions. Total knowledge scores ranged from 0 to 11.The higher scores indicate greater knowledge.

Content validity was established by a panel of six subject-matter experts who independently rated the relevance of every item in both questionnaires on a 4-point scale (1 = not relevant, 4 = highly relevant). For both questionnaires, the item-level content validity index (I-CVI) ranged from 0.83 to 1.00. The universal-agreement scale-level CVI (S-CVI/UA) was 0.86 for HPV Knowledge Questionnaire and 0.82 for HPV Vaccine Knowledge Questionnaire, both exceeding the 0.80 criterion for excellent content validity [48].

#### HPV Vaccination Willingness Scale.

Vaccination willingness was assessed using the HPV Vaccination Willingness Scale developed by Gong Yingjie [49]. This scale is a 5-point Likert scale (ranging from “strongly disagree” to “strongly agree”) comprising three items: (1) I will consider receiving the HPV vaccine; (2) I will definitely receive the HPV vaccine; (3) I will recommend the HPV vaccine to my friends or classmates. The total score of these three items was used to reflect the overall vaccination willingness, with higher scores indicating stronger willingness to receive the HPV vaccine. The Cronbach’s α coefficients of internal consistency of this scale when applied to female non-medical college students were 0.97 [49]. The Cronbach’s α of this scale in this study were 0.87.

### Survey methods

The electronic questionnaire was created using the Wenjuanxing Questionnaire Platform (https://www.wjx.cn). The survey was conducted during class breaks. The investigators first asked the male students to leave the classroom, then explained the purpose of the survey and the inclusion and exclusion criteria for participants to the female students present. Female students who met the inclusion criteria were asked to remain in the classroom, while those who met any exclusion criterion were asked to leave. Subsequently, the investigators explained the requirements for completing the questionnaire to the participants. Once participants had no further questions, the questionnaire was then immediately distributed on-site via a QR (Quick Response) code. Participants voluntarily used their personal mobile phones to scan the code and complete the questionnaire anonymously in one sitting, without any compensation provided for participation. To prevent duplication, each IP address associated with a mobile phone was allowed to submit only once. The questionnaires were collected immediately upon completion. Questionnaires were considered invalid if the personal information was inconsistent, or all options were selected as the same answer.

### Data collection

1293 questionnaires were distributed, and all were collected. In the survey, 102 invalid questionnaires were excluded, leaving 1191 valid questionnaires. The overall valid response rate was 92.1%.

### Statistical analysis

After coding, the collected data were analyzed using descriptive statistics, including frequency, percentage, mean, and standard deviation, on the Chinese version of IBM SPSS 23.0 (IBM Inc., Armonk, NY, USA). Given the large sample size, parametric tests were employed based on the central limit theorem and established literature [50,51] demonstrating the robustness of parametric methods with large samples. Independent sample t-test, ANOVA (Bonferroni correction was used for multiple comparisons within the group), Pearson correlation analysis, and hierarchical multiple regression were used for statistical analysis, and P < .05 was considered statistically significant.

### Ethical considerations

In accordance with the Ethical Considerations, the protocol for this research was approved by the Institutional Review Board at The First Affiliated Hospital of Chengdu Medical College, China. According to Item 2 of Article 32 of the “Ethical Review Measures for Life Sciences and Medical Research Involving Humans” of the People’s Republic of China: “Research conducted using anonymized information data that does not cause harm to humans, involve sensitive personal information, or concern commercial interests may be exempted from ethical review”. All the participants provided written informed consent prior to completing the survey. They were informed of the purpose of the study and they have the right to withdraw from the study at any time. Their survey data were treated to guarantee confidentiality. The study was conducted in accordance with the ethical principles of the Declaration of Helsinki.

## Results

### 1. Participant characteristics

A total of 1191 female undergraduate students from first to third year were included in this study. Their ages ranged from 17 to 25 years. The mean age of the participants was 19.94 ± 1.23 years.For analytical purposes, age was categorized into three groups based on its natural correspondence with academic year within our sample of first- to third-year students: ≤ 19 years (predominantly first-year students), 20–21 years (primarily second- and third-year students), and ≥22 years (older students within this cohort, mainly third-year students reflecting individual circumstances such as later university entrance). The majority of participants were aged 20–21 years (632, 53.1%), followed by ≤19 years (438, 36.8%) and ≥22 years (121, 10.2%). Among the participants, 672 (56.4%) were from healthcare majors, and 519 (43.6%) were from non-healthcare majors; 391 (32.8%) were in their 1st academic year, 474 (39.8%) were in their 2nd academic year, and 326 (32.6%) were in their 3rd academic year. 792 participants had rural residence (66.5%). Regarding socioeconomic indicators, monthly living expenses were categorized into four groups based on the distribution patterns reported in a nationally representative survey [52] of Chinese college students (iiMedia Research, 2024), while ensuring sufficient sample size in each category for meaningful statistical comparison. The categories were: ≤ 1000 CNY, 1001–1500 CNY, 1501–2000 CNY, and >2000 CNY (the latter combining the 2001–3000 CNY and >3000 CNY brackets from the reference survey, which together accounted for approximately 20% of the national student population). In our sample, the largest proportion of students reported monthly living expenses of 1001–1500 CNY (733, 61.5%), followed by 1501–2000 CNY (302, 25.4%), ≤ 1000 CNY (91, 7.6%), and >2000 CNY (65, 5.5%). Most participants (1053, 88.4%) had no sexual history (Table 1).

**Table 1:** Demographic characteristics of study participants (n = 1191).

Variable	Group	Frequency (n)	Percent (%)
**Age**	① ≤ 19	438	36.8
	② 20 ~ 21	632	53.1
	③ ≥ 22	121	10.2
**Academic Major**	Healthcare	672	56.4
	Non- Healthcare	519	43.6
**Academic Year**	① 1^st^ year	391	32.8
	② 2^nd^ year	474	39.8
	③ 3^rd^ year	326	27.4
**Residence**	Urban	399	33.5
	Rural	792	66.5
**Monthly Living Expenses (CNY)**	① ≤ 1000	91	7.6
	② 1001 ~ 1500	733	61.5
	③ 1501 ~ 2000	302	25.4
	④ ＞ 2000	65	5.5
**Sexual History**	Yes	138	11.6
	No	1053	88.4

Note: Currency values are in Chinese Yuan (CNY). At the time of study conduct, the average exchange rate was approximately 1 USD = 7.20 CNY (for reference only).

### 2. Respondents’ HPV knowledge scores and demographic influencing factors

The average score for HPV basic knowledge was 5.21 ± 1.91. The question with the highest accuracy was “Which of the following is the primary way HPV is transmitted?” (85.6%),while the question with the lowest accuracy was “Which age group has a high incidence of HPV infection?” (16.3%).The average score for HPV advanced knowledge was 1.02 ± 1.03. The question with the highest accuracy was “What role does using contraceptive pills play in preventing HPV?” (29.6%), and the question with the lowest accuracy was “What are the other genotypes of HPV that cause cervical cancer in China?” (9.4%). The total score of HPV knowledge was 6.23 ± 2.44 (Table 2).

**Table 2:** HPV knowledge scores of female undergraduates.

Variable	Question	Number of correct answers	Accuracy Rate(%)	Score
**HPV Basic Knowledge**	What is the Chinese name for HPV?	972	81.6	0.82 ± 0.39
	Which kind of HPV can cause cervical cancer?	752	63.1	0.63 ± 0.48
	Which gender(s) can be infected with HPV?	704	59.1	0.59 ± 0.49
	Which age group has a high incidence of HPV infection?	194	16.3	0.16 ± 0.37
	Which of the following is the primary way HPV is transmitted?	1019	85.6	0.86 ± 0.35
	What role does using condoms play in preventing HPV?	959	80.5	0.81 ± 0.40
	How to know if you are infected with HPV?	867	72.8	0.73 ± 0.45
	Who are more likely to get infected with HPV，among the groups with the following conditions?	734	61.6	0.62 ± 0.49
	Total			5.21 ± 1.91
**HPV Advanced Knowledge**	What role does using contraceptive pills play in preventing HPV?	353	29.6	0.30 ± 0.46
	What is the probability of a woman being infected with HPV throughout her lifetime?	243	20.4	0.20 ± 0.40
	What is the probability of developing cervical cancer after being infected with HPV?	139	11.7	0.12 ± 0.32
	What are the most prevalent genotypes of HPV that cause cervical cancer in China?	227	19.1	0.19 ± 0.39
	What are the other genotypes of HPV that cause cervical cancer in China?	112	9.4	0.09 ± 0.29
	What symptoms may occur after HPV infection?	139	11.7	0.12 ± 0.32
	Total			1.02 ± 1.03
**Total HPV Knowledge Scores**				6.23 ± 2.44

This research examined the influence of demographic variables on knowledge of HPV among female undergraduates. The results showed that there were statistically significant differences in the HPV basic knowledge scores among different groups based on age, academic major, academic year, monthly living expenses, and sexual history; statistically significant differences in the HPV advanced knowledge scores among different groups based on age, academic year, and sexual history; statistically significant differences in the total HPV knowledge scores among different groups based on age, academic year, monthly living expenses, and sexual history (P < 0.05) (Table 3).

**Table 3:** HPV knowledge scores of female undergraduates with different demographic characteristics.

Variable		HPV basic knowledge	HPV advanced knowledge	Total HPV knowledge scores
**Age**	① ≤ 19	4.93 ± 2.00	0.93 ± 0.96	5.86 ± 2.48
	② 20 ~ 21	5.39 ± 1.84	1.09 ± 1.07	6.48 ± 2.39
	③ ≥ 22	5.24 ± 1.84	1.00 ± 1.05	6.24 ± 2.43
	F	7.566	3.108	8.388
	P	0.001	0.045	<0.001
	Bonferroni post hoc test	① < ②	① < ②	① < ②
**Academic Major**	Healthcare	5.32 ± 1.90	0.99 ± 1.02	6.30 ± 2.38
	Non- Healthcare	5.06 ± 1.92	1.06 ± 1.06	6.13 ± 2.52
	t	2.272	−1.265	1.240
	P	0.023	0.206	0.215
**Academic Year**	① 1^st^ year	4.88 ± 1.95	0.86 ± 0.94	5.74 ± 2.41
	② 2^nd^ year	5.29 ± 1.89	1.07 ± 1.05	6.35 ± 2.42
	③ 3^rd^ year	5.47 ± 1.85	1.14 ± 1.07	6.61 ± 2.42
	F	9.249	7.581	12.615
	P	<0.001	0.001	<0.001
	Bonferroni post hoc test	① < ②,③	① < ②,③	① < ②,③
**Residence**	Urban	5.35 ± 1.95	1.04 ± 1.05	6.39 ± 2.50
	Rural	5.13 ± 1.89	1.01 ± 1.02	6.14 ± 2.41
	t	1.883	0.515	1.691
	P	0.060	0.607	0.091
**Monthly Living Expenses (CNY)**	① ≤ 1000	4.38 ± 2.24	0.79 ± 0.95	5.18 ± 2.78
	② 1001 ~ 1500	5.22 ± 1.86	1.04 ± 1.04	6.25 ± 2.39
	③ 1501 ~ 2000	5.44 ± 1.87	1.05 ± 1.04	6.49 ± 2.39
	④ > 2000	5.15 ± 1.93	1.02 ± 0.98	6.17 ± 2.43
	F	7.246	1.626	6.902
	P	<0.001	0.182	<0.001
	Bonferroni post hoc test	① < ②,③	–	① < ②,③
**Sexual History**	Yes	5.54 ± 2.02	1.18 ± 1.06	6.72 ± 2.53
	No	5.16 ± 1.90	1.00 ± 1.02	6.16 ± 2.42
	t	2.205	1.980	2.563
	P	0.028	0.048	0.011

### 3. Respondents’ HPV vaccine knowledge scores and demographic influencing factors

The average score for HPV vaccine basic knowledge was 2.99 ± 1.05. The question with the highest accuracy was “Which disease is women primarily vaccinated against HPV to prevent?” (100%),while the question with the lowest accuracy was “Which age group is the target age group recommended for HPV vaccination?” (4.3%).The average score for HPV vaccine advanced knowledge was 2.00 ± 1.53. The question with the highest accuracy was “To what extent can the nine-valent vaccine prevent cervical cancer?” (61.0%), and the question with the lowest accuracy was “Which of the following groups of people can receive HPV vaccine?” (4.9%). The total score of HPV vaccine knowledge was 4.99 ± 2.30 (Table 4).

**Table 4:** HPV vaccine knowledge scores of female undergraduates.

Variable	Quastion	Number of correct answers	Accuracy Rate(%)	Score
**HPV Vaccine Basic Knowledge**	Which disease is women primarily vaccinated against HPV to prevent?	1191	100	1.00 ± 0.00
	Which age group is the target age group recommended for HPV vaccination?	51	4.3	0.04 ± 0.20
	How many times does HPV vaccine need to be administered?	942	79.1	0.79 ± 0.41
	In which of the following situations is the HPV vaccine most effective when administered?	813	68.3	0.68 ± 0.47
	What are the HPV vaccines currently available for vaccination in China?	565	47.4	0.47 ± 0.50
	Total			2.99 ± 1.05
**HPV Vaccine Advanced Knowledge**	To what extent can the vaccination of bivalent or quadrivalent vaccines prevent cervical cancer?	341	28.6	0.29 ± 0.45
	To what extent can the nine-valent vaccine prevent cervical cancer?	727	61.0	0.61 ± 0.49
	Which of the following is the correct understanding of the relationship between receiving the bivalent or quadrivalent vaccine and receiving the nine-valent vaccine?	650	54.6	0.55 ± 0.50
	Which of the following groups of people can receive HPV vaccine?	58	4.9	0.05 ± 0.22
	Which types of HPV that cause cervical cancer can be prevented by vaccination with bivalent or quadrivalent vaccines?	230	19.3	0.19 ± 0.40
	Which types of HPV that cause cervical cancer can be prevented by receiving the nine-valent vaccine?	375	31.5	0.31 ± 0.47
	Total			2.00 ± 1.53
**Total HPV Vaccine Knowledge Scores**				4.99 ± 2.30

This research examined the influence of demographic variables on knowledge of HPV vaccine among female undergraduates. The results showed that there were statistically significant differences in HPV vaccine basic knowledge scores, HPV vaccine advanced knowledge scores, and the total HPV knowledge scores among different groups based on age, academic major, academic year, monthly living expenses, and sexual history (P < 0.05) (Table 5).

**Table 5:** HPV vaccine knowledge scores of female undergraduates with different demographic characteristics.

Variable		HPV vaccine basic knowledge	HPV vaccine advanced knowledge	Total HPV vaccine knowledge scores
**Age**	① ≤ 19	2.77 ± 1.07	1.83 ± 1.50	4.60 ± 2.25
	② 20 ~ 21	3.11 ± 1.04	2.09 ± 1.53	5.21 ± 2.28
	③ ≥ 22	3.17 ± 1.05	2.10 ± 1.58	5.26 ± 2.44
	F	15.835	4.103	9.965
	P	<0.001	0.017	<0.001
	Bonferroni post hoc test	① < ②,③	① < ②	① < ②,③
**Academic Major**	Healthcare	3.03 ± 1.02	2.21 ± 1.58	5.24 ± 2.35
	Non- Healthcare	2.94 ± 1.09	1.73 ± 1.41	4.67 ± 2.21
	t	1.554	5.481	4.287
	P	0.012	<0.001	<0.001
**Academic Year**	① 1^st^ year	2.71 ± 1.05	1.74 ± 1.46	4.45 ± 2.23
	② 2^nd^ year	3.05 ± 1.02	1.94 ± 1.49	4.98 ± 2.22
	③ 3^rd^ year	3.25 ± 1.03	2.40 ± 1.58	5.65 ± 2.34
	F	26.030	17.284	24.971
	P	<0.001	<0.001	<0.001
	Bonferroni post hoc test	①,② < ③ ① < ②	①,② < ③	①,② < ③ ① < ②
**Residence**	urban	3.02 ± 1.04	2.05 ± 1.52	5.07 ± 2.28
	rural	2.98 ± 1.06	1.97 ± 1.53	4.95 ± 2.32
	t	0.623	0.817	0.827
	P	0.533	0.414	0.409
**Monthly Living Expenses (CNY)**	① ≤ 1000	2.58 ± 1.08	1.64 ± 1.55	4.22 ± 2.41
	② 1001 ~ 1500	2.97 ± 1.06	1.98 ± 1.53	4.94 ± 2.31
	③ 1501 ~ 2000	3.17 ± 0.98	2.18 ± 1.51	5.35 ± 2.20
	④ > 2000	3.00 ± 1.10	1.94 ± 1.52	4.94 ± 2.31
	F	7.696	3.204	6.006
	P	<0.001	0.023	<0.001
	Bonferroni post hoc test	① < ②,③ ② < ③	① < ③	① < ②,③
**Sexual History**	Yes	3.25 ± 1.03	2.30 ± 1.50	5.55 ± 2.27
	No	2.96 ± 1.05	1.96 ± 1.53	4.92 ± 2.30
	t	3.131	2.442	3.052
	P	0.002	0.015	0.002

### 4. Respondents’ HPV vaccination willingness and demographic influencing factors

The average scores of the three items on the HPV Vaccination Willingness Scale are all greater than 3.5. The total score of these three items was 11.77 ± 2.19 (Table 6). This research examined the influence of demographic variables on scores of the HPV Vaccination Willingness Scale. The results showed that there are statistically significant differences in the scores of item 1 (I will consider receiving the HPV vaccine), item 2 (I will definitely receive the HPV vaccine), item 3 (I will recommend the HPV vaccine to my friends or classmates) and total scores among different groups based on academic majors and monthly living expenses; statistically significant differences in the scores of item 1, item 2, and total scores among different groups based on residence(P < 0.05) (Table 7).

**Table 6:** HPV vaccination willingness of female undergraduates.

Variable	Item	Score Range	Score
**WILL**_**1**_	I will consider receiving the HPV vaccine	1 ~ 5	4.04 ± 0.79
**WILL**_**2**_	I will definitely receive the HPV vaccine	1 ~ 5	3.85 ± 0.87
**WILL**_**3**_	I will recommend the HPV vaccine to my friends or classmates	1 ~ 5	3.88 ± 0.79
**WILL**_**T**_	–	3 ~ 15	11.77 ± 2.19

Note: WILL_T_ is the total score of variables WILL_1_,WILL_2_, and WILL_3_

**Table 7:** HPV vaccination willingness of female undergraduates with different demographic characteristics.

Variable		WILL_1_	WILL_2_	WILL_3_	WILL_T_
**Age**	① ≤ 19	4.04 ± 0.79	3.85 ± 0.88	3.92 ± 0.77	11.81 ± 2.19
	② 20 ~ 21	4.04 ± 0.79	3.88 ± 0.86	3.88 ± 0.80	11.79 ± 2.20
	③ ≥ 22	4.02 ± 0.78	3.74 ± 0.88	3.76 ± 0.84	11.53 ± 2.17
	F	0.018	1.194	1.940	0.837
	P	0.982	0.303	0.144	0.433
**Academic Major**	Healthcare	4.13 ± 0.78	3.92 ± 0.87	3.96 ± 0.80	12.01 ± 2.17
	Non- Healthcare	3.91 ± 0.79	3.77 ± 0.86	3.79 ± 0.78	11.47 ± 2.18
	t	4.828	2.885	3.671	4.213
	P	<0.001	0.004	<0.001	<0.001
**Academic year**	① 1	4.06 ± 0.79	3.85 ± 0.85	3.89 ± 0.78	11.80 ± 2.17
	② 2	3.99 ± 0.78	3.82 ± 0.88	3.83 ± 0.80	11.64 ± 2.18
	③ 3	4.09 ± 0.79	3.90 ± 0.87	3.95 ± 0.80	11.94 ± 2.23
	F	1.763	0.844	2.499	1.965
	P	0.172	0.430	0.083	0.141
**Residence**	Urban	4.10 ± 0.83	3.96 ± 0.88	3.94 ± 0.83	12.00 ± 2.29
	Rural	4.01 ± 0.77	3.80 ± 0.86	3.85 ± 0.77	11.66 ± 2.13
	t	1.969	3.024	1.723	2.549
	P	0.049	0.003	0.085	0.011
**Monthly Living Expenses (CNY)**	① ≤ 1000	3.68 ± 0.83	3.43 ± 0.82	3.46 ± 0.74	10.57 ± 2.13
	② 1001 ~ 1500	4.02 ± 0.77	3.82 ± 0.86	3.88 ± 0.77	11.72 ± 2.12
	③ 1501 ~ 2000	4.15 ± 0.79	4.01 ± 0.84	3.99 ± 0.81	12.15 ± 2.18
	④ > 2000	4.17 ± 0.82	4.12 ± 0.89	4.03 ± 0.87	12.32 ± 2.44
	F	9.177	13.640	11.344	14.115
	P	<0.001	<0.001	<0.001	<0.001
	Bonferroni post hoc test	① < ②,③,④	① < ②,③,④ ② < ③, ④	① < ②,③,④	① < ②,③,④ ② < ③
**Sexual History**	Yes	3.95 ± 0.901	3.86 ± 0.94	3.82 ± 0.87	11.63 ± 2.47
	No	4.05 ± 0.77	3.85 ± 0.86	3.89 ± 0.78	11.79 ± 2.15
	t	−1.239	0.133	−0.916	−0.730
	P	0.217	0.894	0.361	0.466

### 5. Correlation between knowledge of HPV and the HPV vaccine and vaccination willingness

The scores of individual items and the total score of the HPV Vaccination Willingness Scale were significantly positively correlated with the scores of HPV basic knowledge, HPV advanced knowledge, HPV vaccine basic knowledge, and HPV vaccine advanced knowledge(P < 0.05) (Table 8). Furthermore, we employed a hierarchical multiple regression analysis, treating the total score of the HPV Vaccination Willingness Scale as dependent variables. Demographic characteristics are treated as the first level independent variable, HPV basic knowledge and HPV vaccine basic knowledge as the second level independent variables, and HPV advanced knowledge and HPV vaccine advanced knowledge as the third level variables. The findings revealed that, after controlling for demographic factors, HPV basic knowledge and HPV vaccine basic knowledge jointly explained 12.7% of the variance in the total score of the HPV Vaccination Willingness Scale (ΔR² = 0.127, P < 0.01). HPV vaccine advanced knowledge added a negligible 0.1% (ΔR² = 0.001, P < 0.01), while HPV advanced knowledge did not significantly improve the model (P > 0.05) (Table 9).

**Table 8:** Correlation analysis between HPV knowledge, HPV vaccine knowledge and HPV vaccination willingness (R-value).

Variable	WIIL_1_	WILL_2_	WILL_3_	WILL_T_
**HPV basic knowledge**	0.290**	0.316**	0.292**	0.335**
**HPV advanced knowledge**	0.066*	0.124**	0.115**	0.115**
**Total HPV knowledge scores**	0.255**	0.300**	0.277**	0.311**
**HPV vaccine basic knowledge**	0.283**	0.318**	0.279**	0.329**
**HPV vaccine advanced knowledge**	0.270**	0.300**	0.293**	0.322**
**Total HPV vaccine knowledge scores**	0.308**	0.345**	0.322**	0.364**

Note:Pearson correlation coefficients (R) are reported; significance levels:* P < 0.05, ** P < 0.01.

**Table 9:** Hierarchical multiple regression analysis of female undergraduates’ knowledge scores and HPV vaccination willingness (β value).

Variables	WILL_T_			95%CI
	Step1	Step2	Step3	
**Age**	−0.045	−0.060	−0.052	−0.293 ~ 0.189
**Academic Major**	−0.129**	−0.105**	−0.086**	−0.319 ~ 0.147
**Academic year**	−0.061	−0.006	0.003	−0.197 ~ 0.203
**Residence**	−0.012	−0.007	−0.007	−0.262 ~ 0.248
**Monthly Living Expenses (CNY)**	0.173**	0.131**	0.131**	−0.047 ~ 0.309
**Sexual History**	0.044	0.061**	0.062**	−0.299 ~ 0.423
**HPV basic knowledge**		0.210**	0.180**	0.106 ~ 0.254
**HPV vaccine basic knowledge**		0.206**	0.152**	0.009 ~ 0.295
**HPV advanced knowledge**			−0.035	−0.157 ~ 0.087
**HPV vaccine advanced knowledge**			0.142**	0.044 ~ 0.24
***F* value**	10.157	31.612	27.231	
**R** ^**2**^	0.044	0.171	0.181	
**△R** ^**2**^	−	0.127	0.011	

Note:** P < 0.01.

## Discussion

This study revealed that the knowledge scores of HPV and its vaccine among female undergraduate students in Chengdu were 6.23 ± 2.44 (out of 14) and 4.99 ± 2.30 (out of 11) respectively, both at a relatively low level overall. These results are similar to those reported by Chen et al. [53] in 2022，but lower than those reported by Hu et al. [19] in 2024, possibly due to differences in the assessment methods and difficulty levels of items used to test HPV and HPV vaccine knowledge. Regarding HPV knowledge scores, the HPV basic knowledge score was 5.21 ± 1.91 (out of 8), at a moderate level, while the HPV advanced knowledge score was 1.02 ± 1.03 (out of 6), at a low level. For HPV vaccine knowledge scores, the HPV basic vaccine knowledge score was 2.99 ± 1.05 (out of 5), at a moderate level, whereas the HPV vaccine advanced knowledge score was 2.00 ± 1.53 (out of 6), also at a low level. The disparity in scores between basic and advanced knowledge of HPV and its vaccine may be attributed to two key factors. From the perspective of health communication, basic knowledge is typically disseminated widely through channels such as mass media, campus campaigns, and vaccine promotion, resulting in broad coverage. In contrast, advanced knowledge—often involving medical details—requires systematic learning or professional guidance, resulting in limited coverage. From the audience’s perspective, basic knowledge is generally simple and easy to understand, making it more memorable; whereas advanced knowledge frequently involves complex medical terminology or intricate data, posing a greater difficulty in comprehension. Although healthcare students’ curricula do cover in-depth content on HPV and its vaccines, this material is relatively minor compared to the vast amount of knowledge they are required to learn and be assessed on. Consequently, their mastery of advanced HPV and vaccine-related knowledge may be insufficient or poorly retained.

Female undergraduate students exhibited significant differences in HPV and HPV vaccine knowledge scores across various demographic characteristics. Our study found that students in the 21–22 age group scored significantly higher than those in the ≤ 19 age group across all domains—basic knowledge, advanced knowledge, and total knowledge scores. Similarly, students in their second and third academic year scored significantly higher than first-year students in HPV basic knowledge, HPV advanced knowledge, and total HPV knowledge scores. Moreover, third-year students outperformed both first- and second-year students in HPV basic vaccine knowledge, HPV vaccine advanced knowledge, and total HPV vaccine knowledge scores. These findings align with prior studies [20,21,40–42,54,55], which have consistently shown that knowledge levels tend to be higher among older students or those in more advanced academic years. This pattern largely reflects a “time effect” of knowledge accumulation: knowledge is acquired progressively through life experience and academic exposure over time. Older age implies a longer life course and more opportunities to encounter health-related information, while longer duration in a university setting increases potential exposure to academic lectures, peer discussions, and professional resources. The relatively lower scores among younger and lower-year students further indicate a delayed onset of HPV prevention education. The World Health Organization (WHO) recommends ages 9–14 as the optimal window for HPV vaccination, and ideally, comprehensive education on HPV prevention should begin before adolescence or during middle school. In reality, however, many female students may not receive systematic information about HPV until their later undergraduate years in China. This delay can cause younger and lower-year students to miss the optimal window for preventive decision-making. Therefore, there is an urgent need for higher education institutions to implement systematic and universal HPV prevention education for students early upon entry.

This study found that healthcare majors scored higher than non-healthcare majors in HPV basic knowledge, HPV vaccine basic knowledge, HPV vaccine advanced knowledge, and total HPV vaccine knowledge. Similar findings have been reported in previous research. For instance, medical students demonstrated a higher level of awareness regarding HPV and its vaccine compared to non-medical students [19–23]; biology majors showed greater knowledge of HPV than non-biology majors [12,24,25]; and students in health-related fields exhibited better awareness of HPV and HPV vaccination than those in non-health-related fields [26–30]. Students in medical/biology/health-related majors can acquire knowledge about HPV and its vaccines systematically through specialized coursework. In contrast, students from unrelated majors primarily obtain such information through incidental and fragmentary channels, such as mass media, social media platforms, personal online searches, and other informal sources. These means of information acquisition cannot compare to systematic professional education in terms of the comprehensiveness, accuracy, and depth of knowledge. This further highlights the insufficiency of HPV prevention and health education within higher education institutions.

No statistically significant difference was found in the levels of HPV and HPV vaccine knowledge among students from different residential backgrounds. This finding is consistent with research conducted by Liu et al. [56] in Beijing, China. In contrast, studies by Bencherit et al. [47] in Algeria and Badgujar et al. [57] in Malaysia both indicated that students residing in urban areas had higher levels of HPV and HPV vaccine knowledge than those in rural areas. The difference between China and other developing countries in this regard may be attributed to the greater investment and wider coverage of HPV and vaccine awareness campaigns by the Chinese government, which has effectively mitigated the urban-rural knowledge gap.

This study found that students’ economic status influences their knowledge scores regarding HPV and its vaccine. Specifically, the group with monthly living expenses ≤ 1,000 CNY scored lower than both the 1,001–1,500 CNY and 1,501–2,000 CNY groups. This finding aligns with conclusions from prior research. For instance, a study by Wanderley et al. [38] reported that students who indicated a higher family income achieved higher scores on tests assessing knowledge of HPV, the HPV vaccine, and cervical cancer. Similarly, research by Li et al. [23] found that students with higher mean monthly consumption levels possessed greater knowledge about the HPV vaccine. The “scarcity theory” proposed by Mullainathan and Shafir offers a psychological explanation for this phenomenon. This theory posits that feelings of scarcity influence the way attentional resources are allocated, directing one’s focus predominantly toward issues related to the perceived scarcity [58]. Consequently, students with limited living expenses primarily allocate their attention to concerns related to “financial scarcity”, such as managing their daily budgets. This leaves fewer attentional resources available to focus on, comprehend, and retain preventive health information like that concerning HPV, ultimately resulting in lower knowledge scores on the topic.

Our study found that students with a sexual history scored significantly higher than those without a sexual history across all domains of HPV and HPV vaccine knowledge—basic knowledge, advanced knowledge, and total knowledge scores. This finding is consistent with multiple prior studies [23,26,38]. Sexually active students are more likely to perceive themselves at risk of HPV infection, which may motivate them to actively seek relevant information. Additionally, following sexual debut, they may consult healthcare professionals or discuss topics such as contraception and sexually transmitted infections with partners or close friends. These experiences may indirectly enhance their awareness and understanding of HPV.

Female undergraduate students exhibited significant differences in HPV vaccination willingness across multiple demographic characteristics. Students majoring in healthcare disciplines reported significantly stronger willingness to receive the HPV vaccine compared to those in non-healthcare fields. This aligns with findings from previous studies [31,32]. According to the Health Belief Model, female students in healthcare-related majors, due to their specialized education, can more directly recognize themselves as a potential at-risk group for HPV infection, gain a deeper understanding of the severity of HPV infection, and more clearly perceive the direct benefits of vaccination. In short, healthcare students outperform non-healthcare students in terms of perceived susceptibility, perceived severity, and perceived benefits, leading to a stronger willingness to receive the HPV vaccine.

Furthermore, students from urban areas expressed greater vaccination willingness than those from rural areas, and students with higher monthly living expenses reported stronger willingness than those with lower expenses. These findings are similar to those from prior studies [59,60] conducted in mainland China. In mainland China, household income in rural areas is generally lower than in urban areas, and the total monthly consumption of university students is correlated with their family location and monthly family income [61]. Thus, both urban–rural and living expense disparities can be interpreted as the influence of economic status on students’ decision-making. The “scarcity theory” provides a behavioral economics explanation for this: Poverty induces trade-off thinking [58]. Individuals with limited financial resources operate under tight budgets and are forced to make trade-offs between consumption options. They must constantly weigh opportunity costs when allocating funds. As a result, preventive health expenditures—such as HPV vaccination—are often deprioritized in favor of more immediate needs like food, housing, transportation, or academic expenses. Notably, however, studies from Beijing [56] (the capital of China) and Hong Kong [40], China, reported that family economic status was not a predictor of students’ willingness to receive HPV vaccine. This discrepancy may stem from the high levels of regional economic development in these cities, where most families can afford preventive health services without experiencing financial strain. In addition, lower HPV vaccination willingness among rural students also stems from cultural factors. In Chinese folk tradition, Confucian teachings urge the repression of desires, particularly sexual impulses, to maintain social order [62]. This framework promoted female virginity as vital to family honor and lineage purity [63]. In modern times，societal attitudes toward sexuality have become relatively more liberal in modern times [64]. However, cultural inertia persists, with ideological and societal perceptions lagging behind material changes [65]. Rural areas, due to their relatively underdeveloped economic and cultural environments, tend to maintain more conservative views on sexuality compared to urban centers. HPV is primarily transmitted through sexual contact. Consequently, within the framework of traditional conservative values, HPV is associated with female infidelity [66]; HPV infection has been stigmatized as a “sign of dishonor” [66]; and vaccination is given unnecessary moral overtones [67]. Infection is perceived as a punishment for promiscuous behavior, and the vaccine recipient is associated with uncleanliness, a symbol of defilement [67]. This also contributes to the weaker willingness to receive the HPV vaccine among female university students from rural areas compared to their urban counterparts.

This study found a significant positive association between basic knowledge of HPV and its vaccine and HPV vaccination willingness: the higher the basic knowledge score, the stronger the willingness to vaccinate. Multiple previous research papers [9–14] have also indicated that knowledge about HPV or its vaccine has a positive impact on HPV vaccination willingness.However, advanced knowledge of HPV and its vaccine showed no significant association with vaccination willingness in our sample. This pattern can be effectively explained by the Elaboration Likelihood Model (ELM) proposed by Petty and Cacioppo [68]. The ELM posits that attitude change occurs primarily through two routes: when individuals have sufficient motivation or ability to process persuasive messages, they are more likely to form judgments via the central route processing, involving careful evaluation of message arguments; conversely, when motivation or ability is low, individuals conserve cognitive resources by relying on the peripheral route processing, making judgments based on simple cues rather than deep analysis of issue-relevant content [68]. A key finding from our study provides empirical support for the applicability of the ELM: both healthcare and non-healthcare female undergraduates scored low on HPV and vaccine advanced knowledge. This suggests that, regardless of academic background, female undergraduates generally lack the advanced cognitive resources necessary to engage in central route processing of HPV vaccine information. Consequently, students from both groups are unlikely to activate the central route. Instead, basic knowledge functions as an effective heuristic cue of peripheral route because of its simplicity, clarity, and direct relevance to personal health risk, and is sufficient to drive vaccination willingness. It should be noted that this does not diminish the value of advanced knowledge. As Petty and Cacioppo emphasize, “If the new attitude results from the central route, the attitude is likely to be more enduring than an attitude formed from the peripheral route” [68]. Therefore, if advanced knowledge about HPV and its vaccine could be translated into more accessible, comprehensible formats for the general population, it would facilitate central route processing and thereby more robustly promote both the formation and long-term maintenance of vaccination willingness.

### Limitations

This study has several limitations that should be acknowledged when interpreting its findings. First, this study employed a cross-sectional design, which precludes causal inference regarding the relationship between HPV knowledge and vaccination willingness. Second, the sample was recruited from universities in Chengdu, China. This limits the generalizability of findings to female undergraduates in other regions. Third, vaccination willingness was measured as self-reported intention, not actual vaccination behavior. Social desirability bias may have led participants to overstate their willingness, especially given the preventive and socially endorsed nature of vaccination. Fourth, although the knowledge scale distinguished between basic and advanced items and was reviewed by subject-matter experts, it remains a self-administered questionnaire without objective validation against clinical or behavioral outcomes. Misinterpretation of complex items could introduce measurement error. Fifth, despite controlling for key demographic variables, residual confounding is possible. Other unmeasured factors, such as parental attitudes, could influence both knowledge levels and vaccination intentions. Finally, While our focus on females aligns with WHO’s cervical cancer elimination strategy, we acknowledge that excluding males is a limitation. Notably, HPV vaccination was not approved for males in China when our study was conducted (2024). Future research should include males following this policy change.

## Conclusions

This study reveals that among female undergraduates in Chengdu, China, the level of basic knowledge about HPV and its vaccine is acceptable, whereas the level of advanced knowledge is comparatively low. Demographic factors influencing their knowledge levels include age, academic major, academic year, monthly living expenses, and sexual history. Factors influencing their vaccination willingness include academic major, residence, and monthly living expenses. The study found that higher scores in basic knowledge of HPV and its vaccine are associated with stronger vaccination willingness, suggesting that such foundational information, functioning as an effective peripheral cue within the ELM, boosts vaccination willingness. In contrast, no significant association was observed between advanced knowledge of HPV and HPV vaccine and vaccination willingness，likely because insufficient advanced knowledge prevents students from engaging the central route of the ELM to form stable willingness. To enhance both willingness and actual uptake of the HPV vaccine, disseminating relevant knowledge is a crucial step. Health communication strategies require strengthening and refinement to bridge the knowledge gap across different population groups. Particular emphasis should be placed on improving the accessibility, simplification, and coverage of advanced knowledge of HPV and its vaccine.
